# Echocardiographic assessment of left atrial structure and function in severe aortic stenosis with preserved vs. reduced left ventricular ejection fraction

**DOI:** 10.3389/fcvm.2026.1738702

**Published:** 2026-05-21

**Authors:** Yao Zhang, Yuming Mu

**Affiliations:** 1 Department of Echocardiography, The First Affiliated Hospital of Xinjiang Medical University, Urumqi, China; 2 Xinjiang Key Laboratory of Ultrasound Medicine, Urumqi, China

**Keywords:** aortic stenosis, echocardiography, left atrial function, left atrioventricular coupling index, left ventricular ejection fraction

## Abstract

**Introduction:**

This study investigated the changes in left atrial (LA) structure and function in patients with severe aortic stenosis (AS) by comparing those with preserved left ventricular ejection fraction (LVEF) to those with reduced LVEF.

**Methods:**

Patients with severe AS were classified into two groups: preserved LVEF (≥ 50%) and reduced LVEF (< 50%). Healthy controls were also enrolled. Transthoracic echocardiography and two-dimensional speckle-tracking echocardiography (2D-STE) were employed to measure LA structural parameters and evaluate LA function. Restricted cubic splines (RCS) analyses were used to explore the nonlinear relationship between LVEF and LA parameters.

**Results:**

Patients with an LVEF < 50% had larger LA volumes [LA maximal volume (LAVmax), LA minimal volume (LAVmin), LA pre-systolic volume (LAVpre), and LA volume index (LAVI)] and higher left atrioventricular coupling index (LACI) and LA stiffness index (LASI) than those with an LVEF ≥ 50% (*P* < 0.05). LA functional parameters [including LA total emptying fraction (LATEF), LA active emptying fraction (LAVaEF), LA passive emptying fraction (LAVpEF), LA expansion index (LAEI)] and the absolute values of LA strain were significantly impaired in severe AS patients with an LVEF < 50% compared with those with an LVEF ≥ 50% (*P* < 0.05). Furthermore, the LVEF inflection points for trend changes in LA structure and function were approximately 46% and 59%, respectively.

**Conclusions:**

In patients with severe AS, LA structural and functional impairments are evident even when LVEF ≥ 50%, and become more pronounced in those with an LVEF < 50%.

## 1 Introduction

Aortic stenosis (AS), one of the most common valvular heart diseases clinically, shows particularly higher prevalence in the elderly population (1, 2). In AS patients, chronically elevated left ventricular (LV) afterload accompanies progressive left atrial (LA) enlargement and dysfunction (3). LA volumetric changes reflect the duration and severity of diastolic dysfunction (4); in severe AS, such LA enlargement becomes more pronounced (5, 6). Furthermore, chronic LV pressure overload induces LA remodeling, impairing function across all three phases—reservoir, conduit, and contractile (7). LA enlargement and dysfunction adversely impact clinical outcomes in severe AS. Therefore, evaluating LA structural and functional alterations holds important clinical relevance.

Currently, growing evidence supports the clinical utility of LA strain assessment (8, 9). LA strain is an important complement to volumetric measures; it allows for the assessment of atrial function independently of atrial volume, because it can detect abnormalities in left heart function before atrial dilation occurs, thus offering greater sensitivity (10, 11). Studies have shown that, in the context of elevated LV filling pressure, the incidence of abnormal LA strain is higher than that of abnormal LA volume index (LAVI), and LA strain can identify cardiac dysfunction at an early stage, even before LAVI becomes abnormal (12). This process can be detected using two-dimensional speckle-tracking echocardiography (2D-STE) (8, 13). Atrial strain analysis enables the early detection of atrial structural remodeling and provides deeper insights into LA function, thereby adding incremental value to the structural information provided by conventional echocardiographic parameters.

AS is characterized by progressive valvular narrowing and LV remodeling. Currently, LV decompensation is most commonly diagnosed by characteristic symptoms or a measured LV ejection fraction (LVEF) reduction below 50% (14). Evidence indicates that even mildly reduced LV function, defined as an LVEF of 50%–59%, is associated with adverse outcomes (15, 16).

However, the heterogeneity in LA structure and function between severe AS patients with an LVEF < 50% and those with an LVEF ≥ 50% remains poorly defined. Therefore, this study aims to comprehensively investigate LA structural and functional profiles in severe AS patients stratified by LVEF (< 50% vs. ≥ 50%).

## 2 Methods

### 2.1 Study population

**Figure 1:**
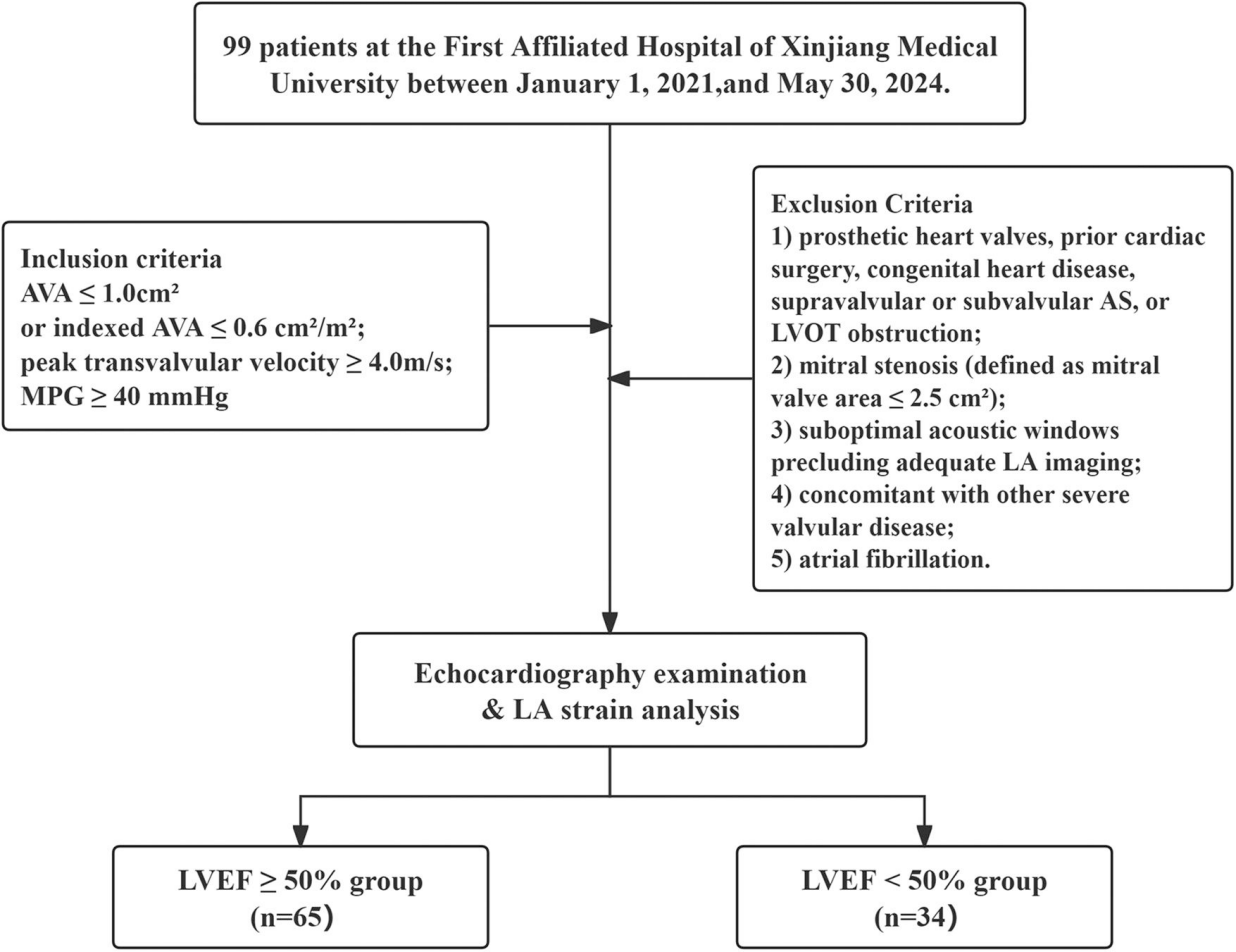
The patients flowchart. LA, left atrial; AVA, aortic valve area; MPG, mean pressure gradient; LVEF, left ventricular ejection fraction; LVOT, left ventricular outflow tract.

A retrospective study was conducted using data from patients diagnosed with severe AS at the First Affiliated Hospital of Xinjiang Medical University between January 1, 2021, and May 30, 2024 (Figure 1). Based on LVEF, patients were stratified into an LVEF ≥ 50% group (*n* = 65; 37 males, 28 females; median age 71.0 years; range 61–92 years) and an LVEF < 50% group (*n* = 34; 18 males, 16 females; median age 71.5 years; range 58–86 years). A separate group of healthy participants served as controls (*n* = 96; 45 males, 51 females; median age 55.5 years; range 49–72 years). This study complied with the requirements of the Declaration of Helsinki and was reviewed and approved by the First Affiliated Hospital of Xinjiang Medical University Medical Ethics Committee (Approval number: K202508-11).

### 2.2 Inclusion and exclusion criteria

Inclusion criteria: Patients meeting the diagnostic criteria for severe AS [defined as aortic valve area (AVA) ≤ 1.0 cm^2^ or indexed AVA ≤ 0.6 cm^2^/m^2^, peak transvalvular velocity ≥ 4.0 m/s, and a mean pressure gradient (MPG) ≥ 40 mmHg (1 mmHg = 0.133 kPa)] were enrolled, per the 2017 joint guidelines for echocardiographic evaluation of AS issued by the European Association of Cardiovascular Imaging (EACVI) and the American Society of Echocardiography (ASE) (17).

Exclusion Criteria: (1) prosthetic heart valves, prior cardiac surgery, congenital heart disease, supravalvular or subvalvular AS, or LV outflow tract (LVOT) obstruction; (2) mitral stenosis (defined as mitral valve area ≤ 2.5 cm^2^); (3) suboptimal acoustic windows precluding adequate LA imaging; (4) concomitant with other severe valvular disease; (5) atrial fibrillation.

### 2.3 Instrumentation

The study utilized a Philips color Doppler ultrasound system equipped with a Q-LAB image analysis workstation and an S5-1 transducer operating at 1–5 MHz, in conjunction with an EchoPAC software workstation.

### 2.4 Echocardiography

#### 2.4.1 LA size and function

According to the ASE/EACVI guidelines (18), the following parameters were measured in apical four- and two-chamber views using the modified biplane Simpson's method: LA maximal volume (LAVmax) at ventricular end-systole, LA minimal volume (LAVmin) at ventricular end-diastole, and LA pre-systolic volume (LAVpre). These measurements were used to calculate:

(1) LA reservoir function parameters:
  Total LA stroke volume (LASV) = LAVmax − LAVmin;
  LA expansion index (LAEI) = [(LAVmax − LAVmin)/LAVmin] × 100%;
  LA total emptying fraction (LATEF) = [(LAVmax − LAVmin)/LAVmax] × 100%.
(2) LA conduit function parameters:
  LA passive emptying volume (LAVp) = LAVmax − LAVpre;
  LA passive emptying fraction (LAVpEF) = (LAVp/LAVmax) × 100%.
(3) LA contractile function parameters:
  LA active emptying volume (LAVa) = LAVpre − LAVmin;
  LA active emptying fraction (LAVaEF) = (LAVa/LAVpre) × 100%.

All parameters were indexed to body surface area.

#### 2.4.2 LV geometry and function

LVEF and LV end-diastolic volume (LVEDV) were measured from apical four- and two-chamber views using the modified biplane Simpson's method (19, 20). Mitral inflow velocities were obtained using pulsed wave Doppler echocardiography to record early diastolic (E) and late diastolic (A) velocities, and the peak early diastolic velocity of the septal and lateral mitral annulus (e′) was measured using pulsed tissue Doppler ultrasound, from which E/A ratio and E/e′ ratio were calculated. The left atrioventricular coupling index (LACI) was defined as (LAVmin/LVEDV) × 100% (21).

#### 2.4.3 2D-STE

**Figure 2:**
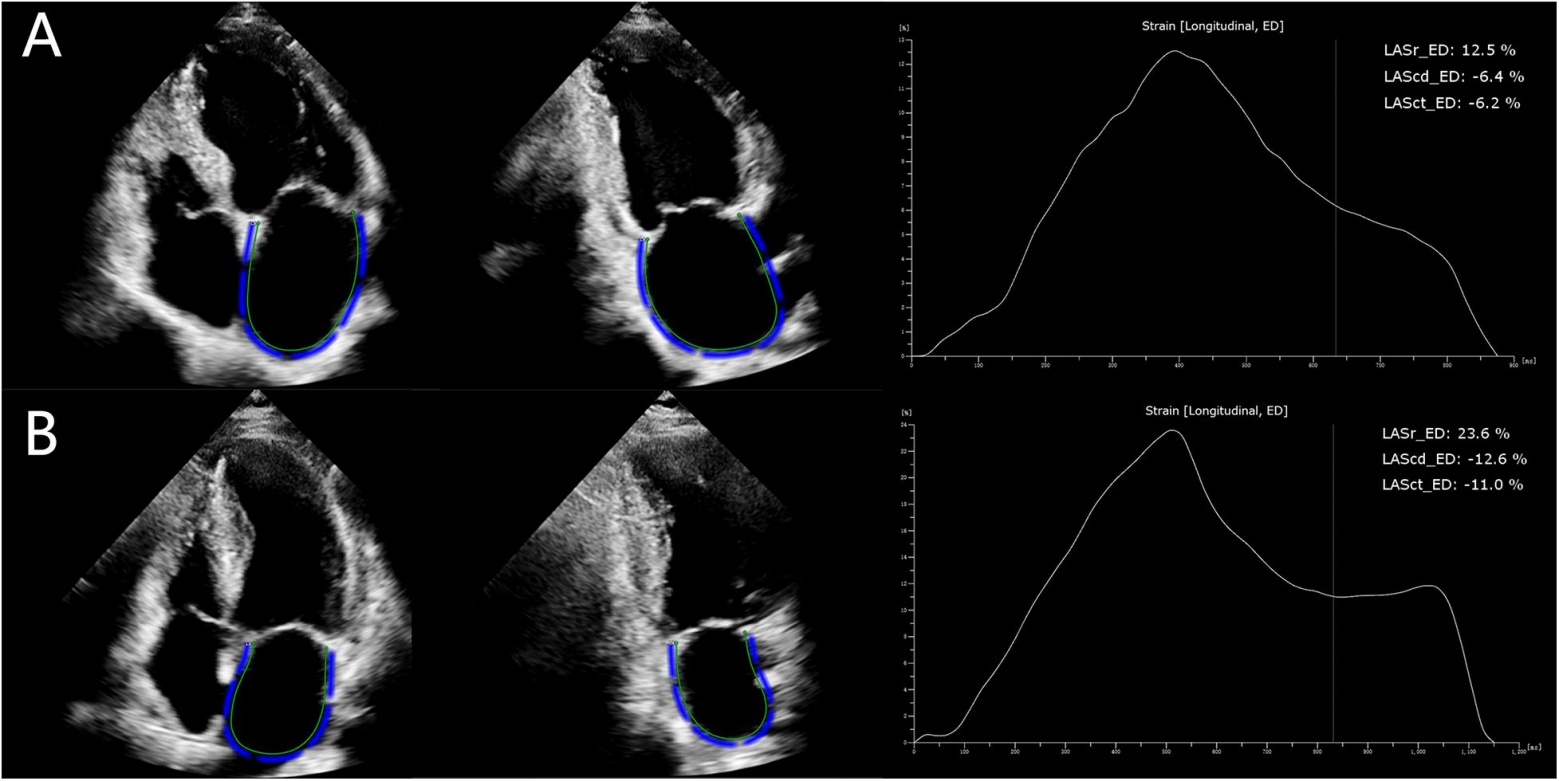
Two-dimensional echocardiographic views of the left atrium (four- and two-chamber views) and three functional phases in severe AS patients with LVEF < 50% **(A)** and those with LVEF ≥ 50% **(B)**. AS, aortic stenosis; LVEF, left ventricular ejection fraction; LASr, left atrial reservoir strain; LASct, left atrial contractile strain; LAScd, left atrial conduit strain.

2D-STE was performed to assess LA strain according to the recommendations of the ASE and the EACVI (18). Apical four- and two-chamber views were acquired to evaluate three phases of LA function (Figure 2): LA reservoir strain (LASr), LA conduit strain (LAScd), LA contractile strain (LASct), and peak atrial longitudinal strain (PALS). The LA stiffness index (LASI) was calculated using the formula: LASI = (E/e′)/LASr, where e′ represents the average of the lateral and septal mitral annular early diastolic velocities. All parameters were derived from the average of three to five consecutive cardiac cycles.

In this study, echocardiographic images were acquired by a certified and professionally trained cardiac sonologist. The data analysis was conducted by two senior sonologists, who were not privy to the patients' medical histories during the analysis. In cases of inconsistent findings, a final decision was reached by consensus.

## 3 Statistical analysis

Statistical analyses were performed using SPSS software (version 26.0) and R software (version 4.4.1). The *χ*^2^ test was used for qualitative data. Quantitative data conforming to normal distribution and homogeneity of variance were expressed as mean ± standard deviations (SD). Comparisons between two independent groups were performed using the *t*-test. One-way analysis of variance (ANOVA) was used for multi-group comparisons, and Least Significant Difference (LSD) method was used for *post-hoc* comparison. Non-normally distributed data were expressed as median with interquartile range [*M* (*P*_25_, *P*_75_)]; the Wilcoxon rank-sum test was used for two-group comparisons, and the Kruskal–Wallis *H* test was applied for multi-group comparisons, with *post-hoc* comparisons performed using the Bonferroni-correction. Error bar plots were generated using the ggplot2 package. Spearman's correlation matrices were visualized through heatmaps created with corrplot package. Restricted cubic splines (RCS) curves were constructed using the rms package, and the optimal knot model was selected based on the Akaike Information Criterion (AIC) minimization principle (Supplementary Table S1). Two-tailed *P* < 0.05 was statistically significant.

## 4 Results

### 4.1 Baseline characteristics

Baseline clinical data included sex, age, heart rate, weight, systolic blood pressure (SBP), diastolic blood pressure (DBP), body mass index (BMI), body surface area (BSA), smoking history, hypertension, coronary heart disease (CHD), chronic kidney disease (CKD), and diabetes. Brain natriuretic peptide (BNP) was significantly higher in the severe AS patients with an LVEF < 50% than those with an LVEF ≥ 50% (*P* < 0.001), as shown in Table 1.

**Table 1:** Severe AS patient characteristics.

Items	LVEF < 50% (*n* = 34)	LVEF ≥ 50% (*n* = 65)	*χ*^2^/Z/*t*	*P* Value
Demographic data				
Gender (male), n (%)	18 (52.9)	37 (56.9)	0.143	0.705
Age, yrs	71.5 (66.8, 77.3)	71.0 (67.0, 80.0)	−0.125	0.900
Heart rate, bpm	77.0 (66.5, 84.3)	73.0 (67.0, 79.0)	−1.280	0.201
SBP, mmHg	118.38 ± 23.31	129.09 ± 20.03	−2.386	0.019
DBP, mmHg	71.41 ± 12.88	72.74 ± 14.95	−0.439	0.662
BMI, kg/m^2^	24.34 (21.72, 26.40)	24.52 (22.96, 27.23)	−0.870	0.385
BSA, m^2^	1.72 (1.60, 1.80)	1.74 (1.65, 1.88)	−1.021	0.307
BNP, pg/mL	4510.00 (2067.50, 13950.00)	1160.00 (438.50, 4190.00)	−4.359	<0.001
Clinical Parameters				
CHD, n (%)	26 (76.5)	44 (67.7)	0.831	0.362
CKD, n (%)	4 (11.8)	5 (7.7)	0.091	0.763
Hypertension, n (%)	18 (52.9)	39 (60.0)	0.367	0.545
Diabetes, n (%)	6 (17.6)	19 (29.2)	1.587	0.208
Smoking, n (%)	7 (20.6)	18 (27.7)	0.597	0.440
Medication				
β-blocker, n (%)	18 (52.9)	29 (44.6)	0.621	0.431
CCB, n (%)	10 (29.4)	26 (40.0)	1.082	0.298
ACEi/ARB, n (%)	12 (35.3)	22 (33.8)	0.022	0.881
Diuretics, n (%)	14 (41.2)	14 (21.5)	4.244	0.039
Statin, n (%)	18 (52.9)	38 (58.5)	0.277	0.599
SGLT2i, n (%)	1 (2.9)	5 (7.7)	0.247	0.619

Values are mean ± SD, *M* (*P*_25_, *P*_75_), n (%).

AS, aortic stenosis; LVEF, left ventricular ejection fraction; SBP, systolic blood pressure, DBP, diastolic blood pressure, BMI, body mass index; BSA, body surface area; BNP, brain natriuretic peptide; CHD, coronary heart disease; CKD, chronic kidney disease; β-blocker, beta receptor blocker; CCB, calcium channel blocker; ACEi, angiotensin-converting enzyme inhibitor; ARB, angiotensin receptor blocker; SGLT2i, sodium-glucose cotransporter 2 inhibitors.

### 4.2 Echocardiographic characteristics

Compared with severe AS patients with an LVEF ≥ 50%, those with an LVEF < 50% exhibited increased Mitral E velocity, E/A ratio and E/e′ ratio, with concomitant decreased Mitral A velocity (*P* < 0.05).

Compared with the control group, both the LVEF ≥ 50% and LVEF < 50% groups exhibited a significantly decreased E/A ratio and increased E/e′ ratio (*P* < 0.05), as shown in Table 2.

**Table 2:** Comparisons of echocardiographic characteristics of patients with severe AS and controls.

Parameters	LVEF < 50% (*n* = 34)	LVEF ≥ 50% (*n* = 65)	Controls (*n* = 96)	*t/Z/H*	*P* Value
Peak velocity, m/s	4.85 ± 0.78	5.14 ± 0.75		−1.766	0.081
AVA, cm^2^	0.53 ± 0.23	0.54 ± 0.18		−0.426	0.671
Indexed AVA, cm^2^/m^2^	0.26 (0.21,0.42)	0.30 (0.24,0.37)		−0.777	0.437
MPG, mmHg	58.36 ± 16.84	64.28 ± 21.76		−1.382	0.170
Mitral E velocity, m/s	0.91 (0.68, 1.21)^†^	0.73 (0.59, 0.91)*	0.81 (0.75, 0.87)	11.453	0.003
Mitral A velocity, m/s	0.72 (0.46, 1.10)^†^	0.98 (0.81, 1.12)*	0.62 (0.54, 0.71)	51.792	<0.001
E/A ratio	0.83 (0.57, 1.70)^*,†^	0.71 (0.56, 0.81)*	1.31 (1.18, 1.54)	67.615	<0.001
E/e′ ratio	18.84 (13.79, 23.84)*	15.63 (10.84, 19.14)*	7.14 (6.64, 7.70)	122.983	<0.001

Values are mean ± SD, *M* (*P*_25_, *P*_75_).

*P *<* 0.05 vs. controls; ^†^*P* *<* 0.05 vs. LVEF ≥ 50% group.

AS, aortic stenosis; LVEF, left ventricular ejection fraction; AVA, aortic valve area; MPG, mean pressure gradient.

### 4.3 LA phasic volumes and function parameters

**Figure 3:**
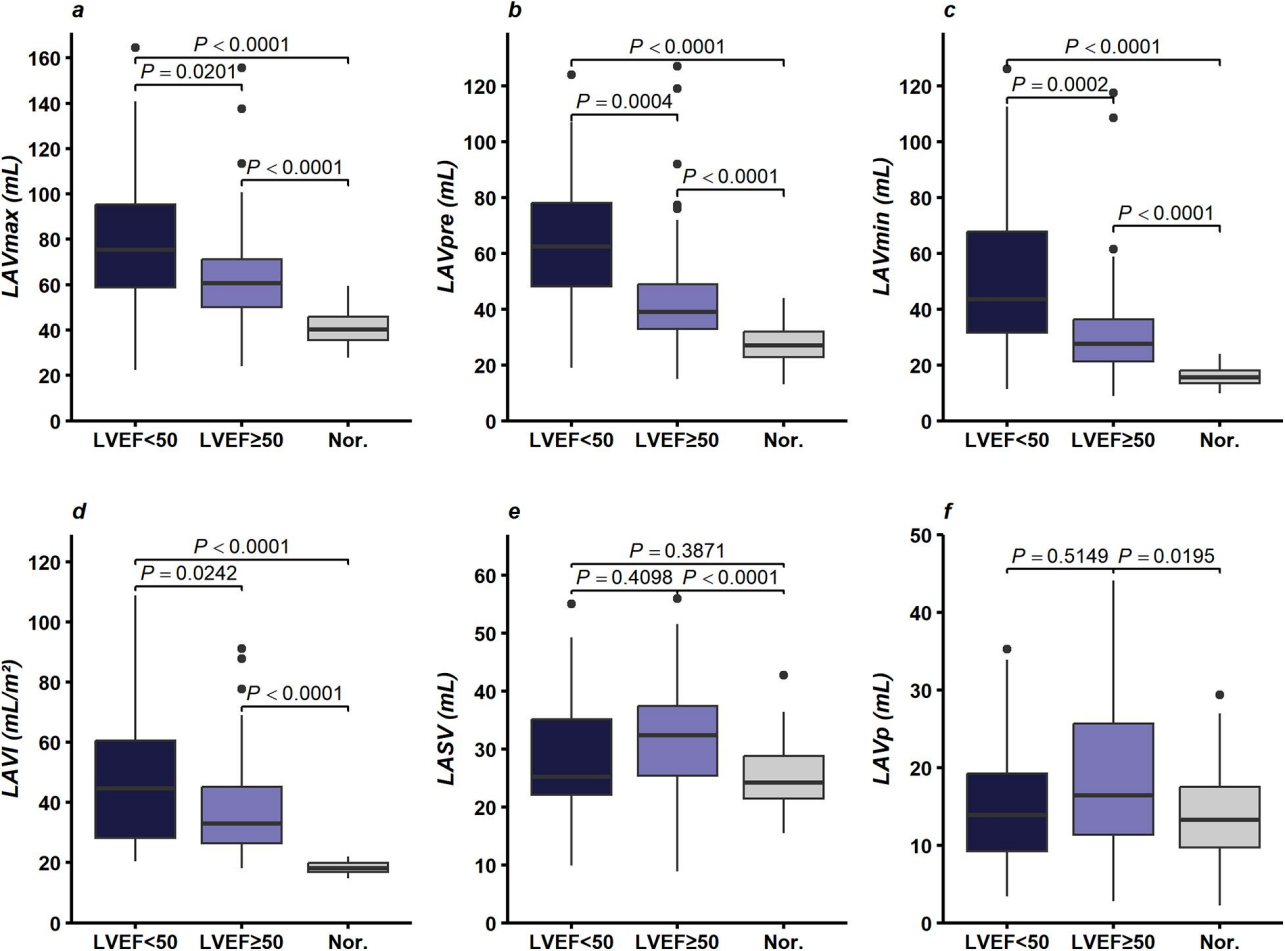
Comparison of LA structural parameters in severe AS patients stratified by LVEF (< 50% vs. ≥ 50%) and controls: **(a)** LAVmax (mL), **(b)** LAVpre (mL), **(c)** LAVmin (mL), **(d)** LAVI (mL/m²), **(e)** LASV (mL), and **(f)** LAVp (mL). Abbreviations: AS = Aortic stenosis; LVEF = Left ventricular ejection fraction; LA = Left atrial; LAVmax = Left atrial maximal volume; LAVpre = Left atrial pre-systolic volume; LAVmin = Left atrial minimal volume; LAVI = Left atrial volume index; LASV = Left atrial stroke volume; LAVp = LA passive emptying volume.

LAVmax, LAVmin, LAVpre and LAVI in severe AS patients with an LVEF < 50% were higher than those with an LVEF ≥ 50%, with patients in both LVEF ≥ 50% and LVEF < 50% groups exhibiting higher values than the control group (*P* < 0.05), as shown in Figure 3 and Supplementary Table S2.

**Figure 4:**
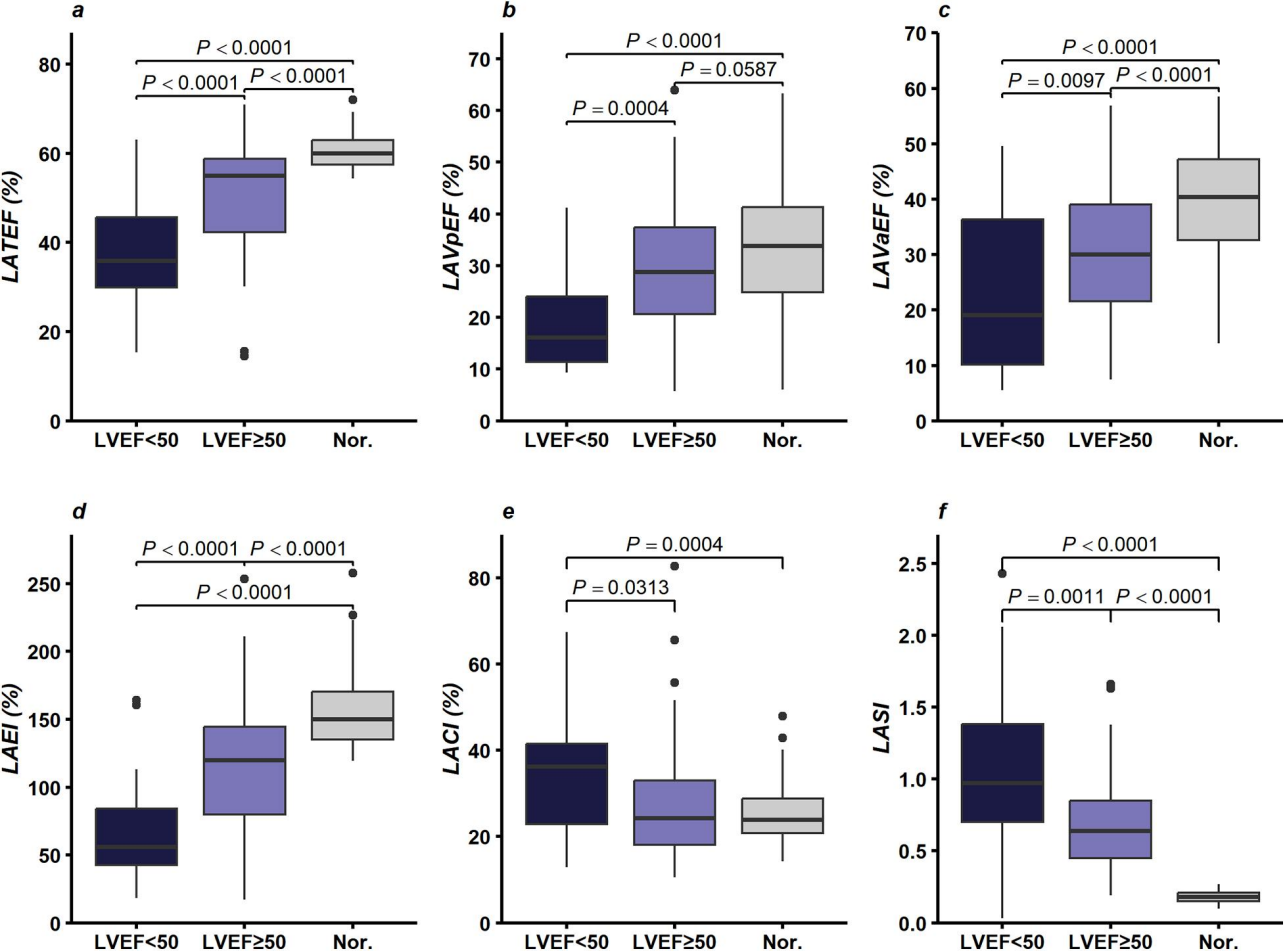
Comparison of LA functional parameters in severe AS patients stratified by LVEF (< 50% vs. ≥ 50%) and controls: **(a)** LATEF (%), **(b)** LAVpEF (%), **(c)** LAVaEF (%), **(d)** LAEI (%), **(e)** LACI (%), and **(f)** LASI. Abbreviations: LA = Left atrial; AS = Aortic stenosis; LVEF = Left ventricular ejection fraction; LATEF = Left atrial total emptying fraction; LAVaEF = Left atrial active emptying fraction; LAVpEF = Left atrial passive emptying fraction; LAEI = Left atrial expansion index; LASI = Left atrial stiffness index; LACI = Left atrioventricular coupling index.

Regarding volumetric parameters of LA function, LATEF, LAVaEF, LAVpEF, and LAEI in those with an LVEF < 50% were lower than those with an LVEF ≥ 50% (*P* < 0.05), with both groups showing lower values than the control group (*P* < 0.05). Conversely, LACI and LASI were higher in patients with an LVEF < 50% compared with those with an LVEF ≥ 50% (*P* < 0.05), with both groups exhibiting higher values than the control group (*P* < 0.05), as shown in Figure 4 and Supplementary Table S2.

### 4.4 LA strain parameters

**Figure 5:**
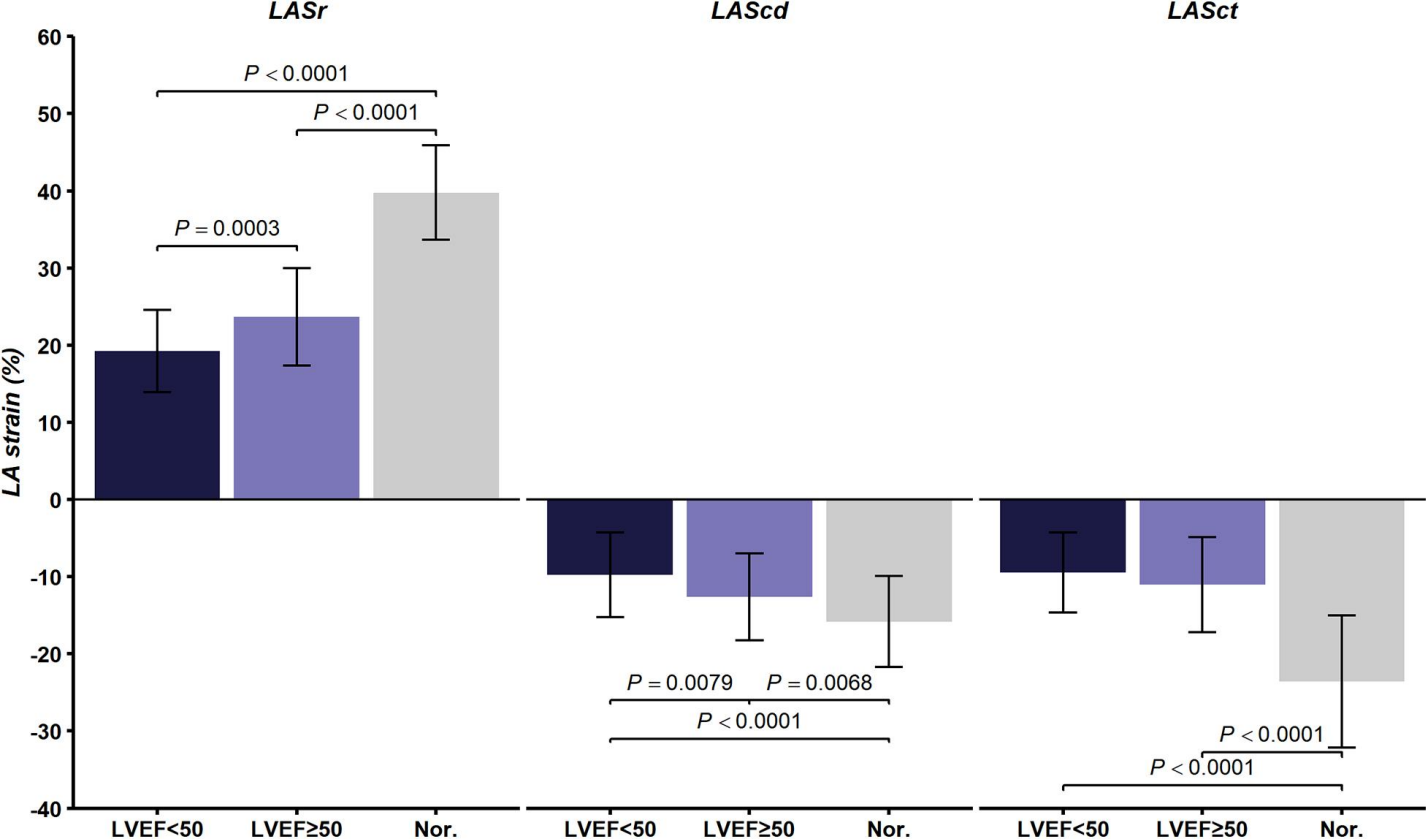
Comparison of LA strain parameters in severe AS patients stratified by LVEF (< 50% vs. ≥ 50%) and controls, mean ± SD. LA, left atrial; AS, aortic stenosis; LVEF, left ventricular ejection fraction; LASr, left atrial reservoir strain; LASct, left atrial contractile strain; LAScd, left atrial conduit strain; PALS, peak atrial longitudinal strain.

The absolute values of LASr, LASct, LAScd, and PALS were lower in severe AS patients with an LVEF < 50% than those with an LVEF ≥ 50% (*P* < 0.05). Both patient groups exhibited lower values than the control group (*P* < 0.05). Notably, LA systolic strain decreased more markedly, as shown in Supplementary Table S3. The differences in LA strain parameters among the three groups are illustrated in Figure 5.

### 4.5 Associations between LVEF and LA remodeling in patients with severe AS

**Figure 6:**
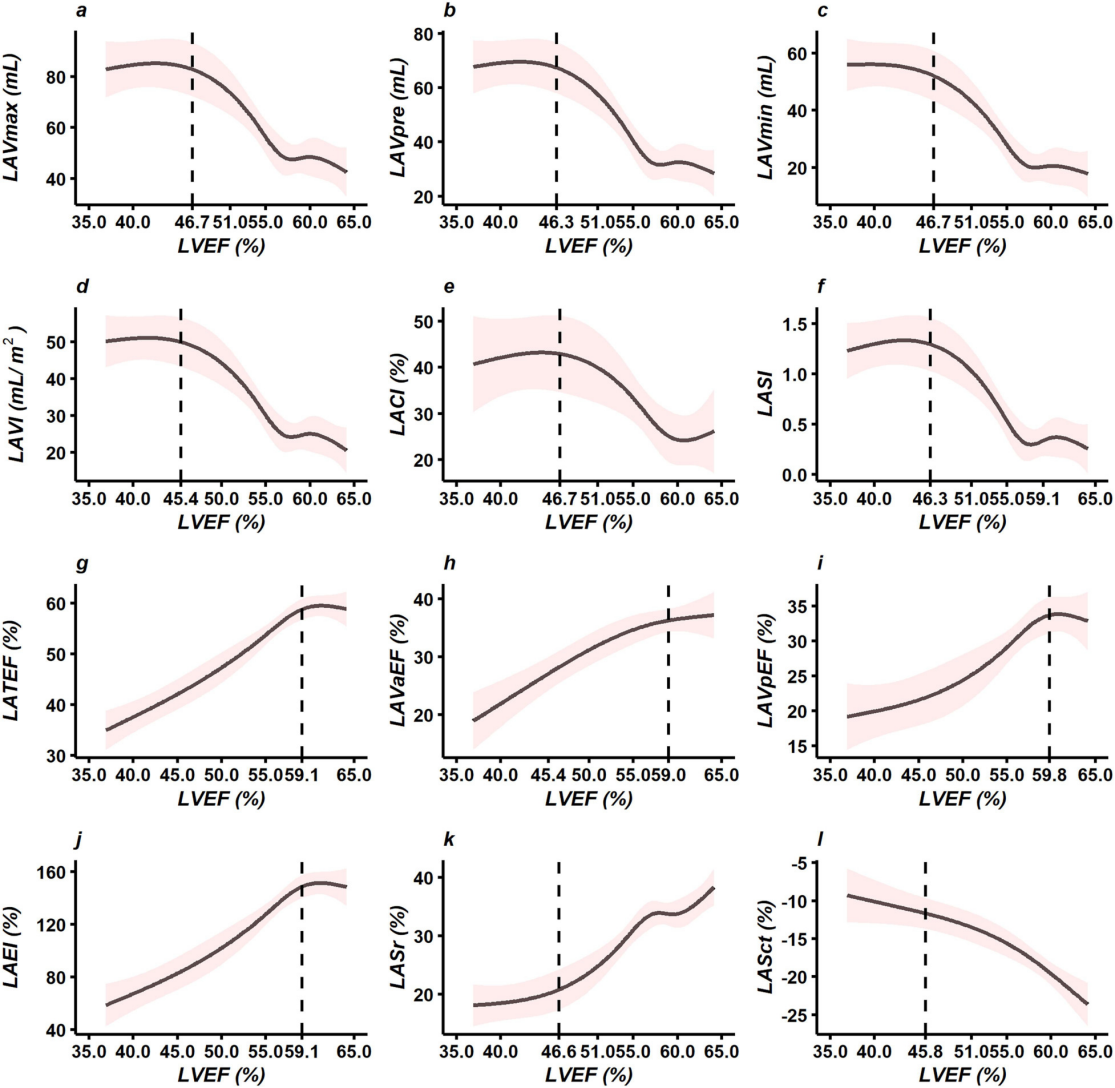
RCS analyses revealed nonlinear associations between LVEF and LA echocardiographic parameters in patients with severe AS: **(a)** LAVmax (mL), **(b)** LAVpre (mL), **(c)** LAVmin (mL), **(d)** LAVI (mL/m²), **(e)** LACI (%), **(f)** LASI, **(g)** LATEF (%), **(h)** LAVaEF (%), **(i)** LAVpEF (%), **(j)** LAEI (%), **(k)** LASr (%), and **(l)** LASct (%). Abbreviations: RCS = Restricted cubic splines; LA = Left atrial; AS = Aortic stenosis; LVEF = Left ventricular ejection fraction; LAVmax = Left atrial maximal volume; LAVpre = Left atrial pre-systolic volume; LAVmin = Left atrial minimal volume; LAVI = Left atrial volume index; LATEF = Left atrial total emptying fraction; LAVaEF = Left atrial active emptying fraction; LAVpEF = Left atrial passive emptying fraction; LAEI = Left atrial expansion index; LASI = Left atrial stiffness index; LACI = Left atrioventricular coupling index; LASr = Left atrial reservoir strain; LASct =Left atrial contractile strain.

In patients with severe AS, RCS analyses revealed nonlinear associations between LVEF and LA parameters, as shown in Figure 6.

LA structural parameters (including LAVmax, LAVpre, LAVmin, and LAVI) decreased with increasing LVEF. Beyond inflection points at LVEF of 46.7%, 46.3%, 46.7%, and 45.4%, respectively, the rate of decline accelerated markedly (Figures 6a–d). LA function parameters (including LATEF, LAVaEF, LAVpEF, and LAEI) increased with LVEF. Beyond inflection points at LVEF of 59.1%, 59.0%, 59.8%, and 59.1%, respectively, the rate of increase slowed (Figures 6g–j).

Conversely, LACI and LASI decreased with increasing LVEF, with a more pronounced decline beyond inflection points at LVEF of 46.7% and 46.3%, respectively (Figures 6e,f).

LA strain parameters, including the absolute values of LASr and LASct, increased with LVEF and exhibited a steeper rise beyond inflection points at LVEF of 46.6% and 45.8%, respectively (Figures 6k,l).

### 4.6 Correlation analysis of echocardiographic parameters in patients with severe AS

**Figure 7:**
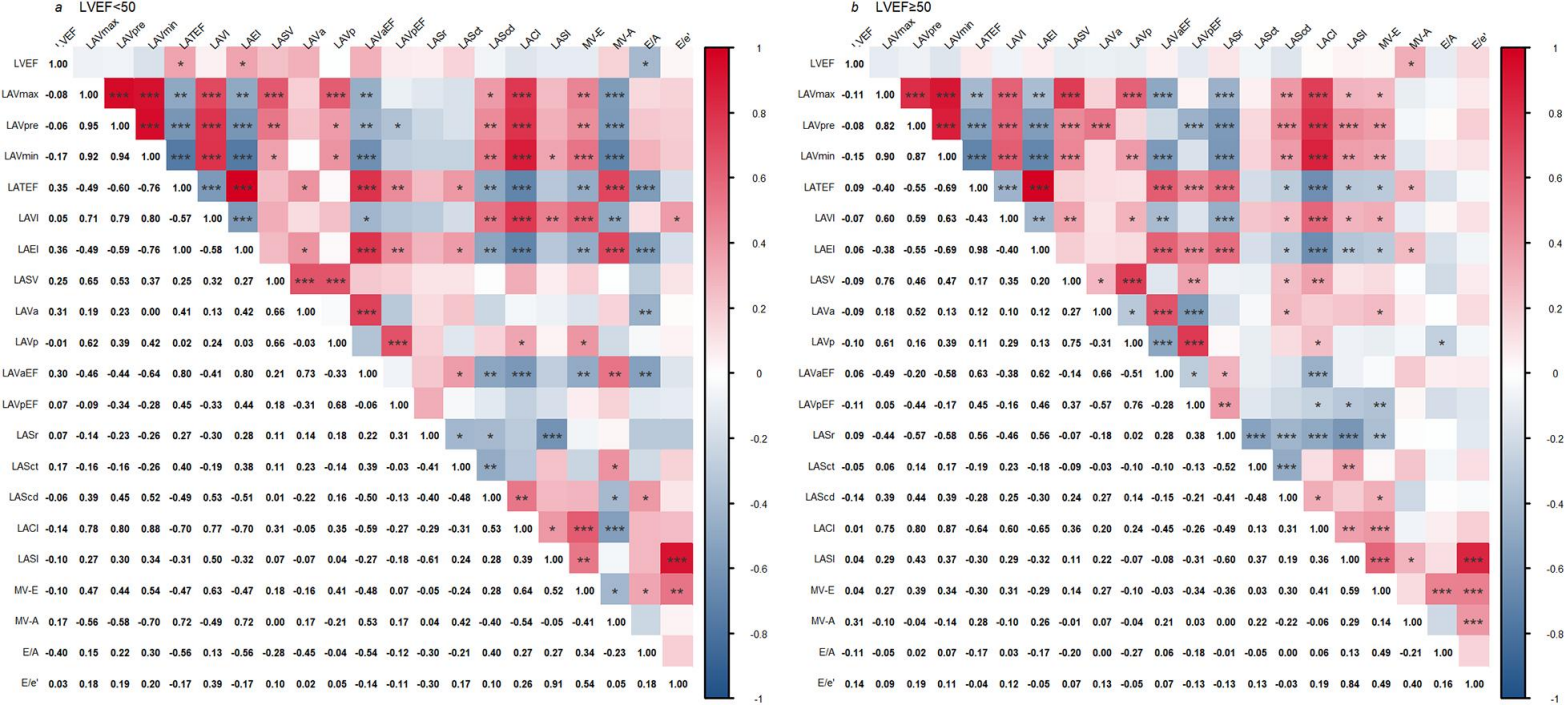
Correlation analysis of echocardiographic parameters in patients with severe AS. **(a)** Heat maps of parameter correlations in patients with an LVEF < 50%; **(b)** Heat maps of parameter correlations in patients with an LVEF ≥ 50%. Color shading for each cell uses a red-blue gradient for the magnitude and direction of the correlation coefficient (red: high/positive correlation, blue: low/negative correlation), and the value within the cell represents the bivariate correlation coefficient. * *P* < 0.05; ** *P* < 0.01; *** *P* < 0.001, abbreviations are defined in the List of Abbreviations.

In severe AS patients with an LVEF < 50%, LVEF was positively correlated with LAEI and LATEF (r = 0.36, *P* < 0.05; r = 0.35, *P* < 0.05) and negatively correlated with E/A (r = −0.40, *P* < 0.05). LAVI was positively correlated with MV-E and E/e′ (r = 0.63, *P* < 0.001; r = 0.39, *P* < 0.05) and negatively correlated with MV-A (r = −0.49, *P* < 0.01), as shown in Figure 7a.

In severe AS patients with an LVEF ≥ 50%, MV-E was negatively correlated with LAVpEF, LATEF, LAEI, and LASr (r = −0.34, *P* < 0.01; r = −0.30, *P* < 0.05; r = −0.29, *P* < 0.05; r = −0.36, *P* < 0.01) and positively correlated with LAScd, LAVI, LAVa, LACI, and LASI (r = 0.30, *P* < 0.05; r = 0.31, *P* < 0.05; r = 0.27, *P* < 0.05; r = 0.41, *P* < 0.001; r = 0.59, *P* < 0.001). E/e′ was positively correlated with LASI (r = 0.84, *P* < 0.001), as shown in Figure 7b.

## 5 Discussion

AS is a common valvular heart disease. This study evaluated LA structural and functional parameters in patients with severe AS by comparing those with preserved LVEF to those with reduced LVEF. We found that severe AS substantially affected LA structure and function regardless of LVEF (< 50% or ≥ 50%), manifesting as structural remodeling (e.g., increased volume index) and functional impairment. Even severe AS patients with preserved or mildly reduced LVEF exhibited LA remodeling and morphological alterations. The LVEF < 50% group demonstrated larger LA dimensions, greater volume parameters, and more severe dysfunction, along with impaired LA reservoir, conduit, and contractile functions.

The LA serves as a critical buffer chamber before blood enters the left ventricle; changes in its volume reflect long-term increases in LV filling pressure. Compared with the LA diameter, LA volume provides a more accurate assessment of atrial function (22, 23). LA enlargement, the most common macrostructural alteration (24), is particularly pronounced in patients with severe AS. According to the AS staging criteria, Stage 2 is characterized by an increased LAVI (> 34 mL/m^2^), while Stages 3 and 4 are characterized by a further increase in LAVI (25). As a volume-derived measure, LAVI is less influenced by age and offers high comparability. Furthermore, as a key indicator of left heart impairment, increased LAVI reflects worsening AS severity and indicates early diastolic dysfunction in patients with severe AS (26). Rusinaru et al. (7) established that larger LAVI correlates with lower LVEF, further demonstrating its association with AS severity. Consequently, assessment of LAVI is of significant clinical importance for identifying left heart damage in patients with severe AS.

This study demonstrated increased LAVmax, LAVmin, LAVpre, and LAVI in severe AS patients with an LVEF < 50% compared with those with an LVEF ≥ 50%. The RCS results indicated that the aforementioned LA structural parameters exhibited a nonlinear correlation with LVEF. As LVEF decreased, when LVEF was greater than approximately 46%, LA parameters increased significantly with decreasing LVEF; once LVEF fell below this threshold, the rate of increase in LA parameters with decreasing LVEF gradually slowed. For patients with LVEF less than approximately 46%, even if the rate of increase in LA enlargement appeared to slow, this did not indicate clinical improvement; rather, it suggested that LA function had been severely impaired.

LA function can be classified into reservoir, conduit, and contractile functions based on the cardiac phase. This study demonstrated that, compared with the group with an LVEF ≥ 50%, the LA reservoir function indices (LAEI, LATEF) were significantly reduced in those with an LVEF < 50%, and these values decreased further as LVEF declined. Additionally, both LA conduit function (LAVpEF) and LA contractile function (LAVaEF) were significantly impaired, with the reduction being more pronounced in those with an LVEF < 50% compared with those with an LVEF ≥ 50%.

The RCS results indicated that, in patients with severe AS, LA functional parameters (LATEF, LAVaEF, LAVpEF, LAEI) exhibited a nonlinear correlation with LVEF. Specifically, when LVEF was greater than approximately 59%, the decline in these parameters with decreasing LVEF was relatively gradual; however, when LVEF was less than approximately 59%, the decline was steeper. Particular attention should be paid to patients with LVEF between 50% and 59%, as signs of LA dysfunction were already evident even though LVEF exceeded 50%.

LA strain is a sensitive indicator for assessing LA systolic and diastolic function. Although LA strain is influenced by LV structural and functional abnormalities, changes in LA strain typically occur prior to alterations in LV function or structure (27). Studies showed that in valvular heart disease, the absolute values of atrial strain parameters (during the reservoir, conduit, and contractile phases) were significantly reduced, suggesting LA dysfunction (28). Among these, LASr was considered a sensitive marker for identifying overall LA dysfunction (29, 30). Compared with structural parameters, LA strain parameters more directly reflected LA functional characteristics. In addition, multiple studies confirmed the prognostic value of LA strain for adverse events in AS (31–34).

The results of this study indicated that the absolute values of LASr, LASct, and LAScd decreased more significantly in severe AS patients with an LVEF < 50% compared with those with an LVEF ≥ 50%, with the decrease in LASct more marked. Ferreira et al. (35) also demonstrated impaired LA function at all stages in severe AS patients, which was similar to the results of the present study, but their study was limited to severe AS patients with normal LVEF. Consequently, this study further demonstrated that severe AS patients with an LVEF < 50% exhibited more severe impairment of LA function. Consequently, even when LVEF was preserved, patients with severe AS already exhibited significant impairment in LA strain. Therefore, assessing atrial function is helpful in detecting cardiac abnormalities.

Previous studies have reported that LA dysfunction usually precedes LA enlargement, suggesting that structural and functional changes may progress asynchronously (36, 37). However, given that this study focused on patients with severe AS, the study demonstrated that LA structural parameters were increased and functional parameters were reduced in patients with severe AS regardless of LVEF status (< 50% or ≥ 50%); these changes were more pronounced in patients with an LVEF < 50%.

In valvular heart disease, LA dysfunction or left atrial-ventricular dyssynchrony can impair overall cardiac function, ultimately leading to heart failure. As a measure that integrates LA morphological and functional parameters, LACI not only quantifies the severity of LV diastolic dysfunction but also reflects LA structural and functional characteristics, thereby aiding in the assessment of LA function (38). Regarding changes in LACI, we observed that the values increased more significantly in severe AS patients with an LVEF < 50% compared with those with an LVEF ≥ 50%, indicating that changes in LA volume were more pronounced relative to changes in LV volume. The more severe the imbalance between LA and LV volumes at end-diastole, the more severe the impairment in atrioventricular coupling (39), suggesting cardiac dysfunction or structural changes. Moreover, LASI served as a reliable indicator in patients with severe AS, and LASI also showed a similar increasing trend as LACI, consistent with the findings of Alfuhied et al. (40), who demonstrated increased LASI values in patients with severe AS.

## 6 Limitations

- As a single-center study, potential selection bias may exist.
- The average age in the control group was lower than that in the group of patients with severe AS because of the very limited number of healthy elderly volunteers.
- Echocardiography and 2D-STE are subject to inherent limitations, particularly the latter's limitation to tracking speckle motion within a 2D plane and its reliance on accurate endocardial border delineation and image quality.
- Patients with AS frequently present with comorbidities such as hypertension, coronary artery disease, and diabetes, often requiring multiple medications. These factors may act as confounding variables in our data analysis.
- The sample size of severe AS patients with an LVEF < 50% was limited, warranting a larger sample size for validation in future studies.
- In this study, dobutamine stress echocardiography was not performed, since all AS patients exhibited an MPG ≥ 40 mmHg in the severe range and were considered high-risk.
- This is a cross-sectional study. In the future, long-term follow-up studies are needed to assess the prognostic value of LA parameters.

## 7 Conclusions

In patients with severe AS, LA structure and function are significantly affected regardless of LVEF being preserved or reduced. Compared with patients with preserved LVEF, those with reduced LVEF exhibited significantly increased LA structural parameters and significant impairment in LA reservoir, conduit, and contractile functions. Furthermore, the LVEF inflection points for trend changes in LA structure and function were approximately 46% and 59%, respectively, but the clinical utility of the aforementioned thresholds requires further validation in longitudinal studies.

## Data Availability

The original contributions presented in the study are included in the article/Supplementary Material, further inquiries can be directed to the corresponding author.
