# Supplementary material for: Echocardiographic assessment of left atrial structure and function in severe aortic stenosis with preserved vs. reduced left ventricular ejection fraction
Source: Front Cardiovasc Med. 2026 May 21;13:1738702. doi: 10.3389/fcvm.2026.1738702 (PMC13233431; doi:10.3389/fcvm.2026.1738702)
Supplement: Supplementary file 1 [file Table1.docx]

| **Table S1. AIC values for restricted cubic spline with different knot numbers.** | | | | | |
| --- | --- | --- | --- | --- | --- |
| Number of Knots | 3 | 4 | 5 | 6 | 7 |
| LAVmax | 1851.890 | 1849.788 | 1848.867 | 1850.249 | 1852.436 |
| LAVpre | 1810.017 | 1806.238 | 1804.976 | 1806.223 | 1808.184 |
| LAVmin | 1781.956 | 1778.812 | 1778.354 | 1779.775 | 1781.388 |
| LAVI | 1682.593 | 1680.557 | 1678.879 | 1678.741 | 1679.718 |
| LASV | 1399.237 | 1400.875 | 1401.303 | 1402.929 | 1404.707 |
| LAVp | 1365.743 | 1367.486 | 1369.389 | 1371.366 | 1373.265 |
| LATEF | 1443.453 | 1441.549 | 1442.670 | 1444.134 | 1443.307 |
| LAVaEF | 1542.460 | 1544.066 | 1545.971 | 1547.574 | 1546.624 |
| LAVpEF | 1523.010 | 1521.891 | 1523.336 | 1525.048 | 1526.014 |
| LACI | 1830.016 | 1828.626 | 1829.525 | 1831.452 | 1833.492 |
| LAEI | 2001.324 | 1998.925 | 1999.696 | 2001.035 | 1998.038 |
| LASI | 421.855 | 417.917 | 415.334 | 416.253 | 418.250 |
| LASr | 1415.799 | 1416.472 | 1414.675 | 1416.107 | 1418.298 |
| LASct | 1411.710 | 1413.236 | 1413.603 | 1415.255 | 1416.431 |
| LAScd | 1263.571 | 1262.829 | 1264.149 | 1265.684 | 1268.175 |
| Abbreviations: AIC = Akaike information criterion; LAVmax = Left atrial maximal volume; LAVpre = Left atrial pre-systolic volume; LAVmin = Left atrial minimal volume; LAVI = Left atrial volume index; LASV = Left atrial stroke volume; LAVp = Left atrial passive emptying volume; LATEF = Left atrial total emptying fraction; LAVaEF = Left atrial active emptying fraction; LAVpEF = Left atrial passive emptying fraction; LACI = Left atrioventricular coupling index; LAEI = Left atrial expansion index; LASI = Left atrial stiffness index; LASr = Left atrial reservoir strain; LASct =Left atrial contractile strain; LAScd = Left atrial conduit strain. | | | | | |
