# Supplementary material for: Echocardiographic assessment of left atrial structure and function in severe aortic stenosis with preserved vs. reduced left ventricular ejection fraction
Source: Front Cardiovasc Med. 2026 May 21;13:1738702. doi: 10.3389/fcvm.2026.1738702 (PMC13233431; doi:10.3389/fcvm.2026.1738702)
Supplement: Supplementary file 2 [file Table2.docx]

| **Table S2. LA echocardiographic **parameters in** patients with severe AS and controls**,** mean *±* SD*/M (P*_25_*, P*_75_*)*** | | | | | |
| --- | --- | --- | --- | --- | --- |
|  | LVEF < 50%  (n = 34) | LVEF ≥ 50%  (n = 65) | Controls  (n = 96) | *F/H* | *P* Value |
| LAVmax, mL | 75.50 (58.00,97.50)^*^ | 60.70 (49.85, 72.45)^*^ | 40.20 (34.98, 45.88) | 78.306 | < 0.001 |
| adjusted^a^ | 74.42 (62.14,86.69) | 59.04 (53.85, 64.24) | 41.03 (37.70, 44.35) | 20.383 | < 0.001 |
| LAVmin, mL | 43.65 (31.10,69.35)^*,†^ | 27.60 (20.75, 36.65)^*^ | 15.50 (13.40, 18.15) | 103.921 | < 0.001 |
| adjusted^a^ | 43.00 (29.14,56.85) | 27.25 (23.84, 30.65) | 16.23 (13.92, 18.54) | 15.653 | < 0.001 |
| LAVpre, mL | 62.50 (47.50,78.75)^*,†^ | 39.00 (33.00, 50.00)^*^ | 27.00 (22.25, 32.00) | 83.234 | < 0.001 |
| adjusted^a^ | 61.93 (50.91,72.94) | 38.72 (34.14, 43.29) | 27.82 (25.96, 29.68) | 21.530 | < 0.001 |
| LAVI, mL/m² | 44.74 (27.92,60.97)^*^ | 32.98 (26.12, 46.56)^*^ | 18.10 (16.86, 19.90) | 136.178 | < 0.001 |
| adjusted^a^ | 44.91 (33.50,56.31) | 32.66 (28.21,37.12) | 18.62 (17.70,19.53) | 26.123 | < 0.001 |
| LASV, mL | 25.25 (21.60,35.30) | 32.40 (25.35, 37.65)^*^ | 24.20 (21.35, 28.80) | 23.099 | < 0.001 |
| adjusted^a^ | 23.96 (18.58,29.35) | 30.31 (27.63,33.00) | 25.92 (23.85,27.99) | 3.834 | 0.023 |
| LAVa, mL | 11.55 (6.22,18.23) | 12.04 (8.10, 16.85) | 10.40 (8.00, 14.10) | 2.336 | 0.311 |
| adjusted^a^ | 12.31 (8.41,16.21) | 11.82 (9.22,14.42) | 10.19 (8.59,11.80) | 0.603 | 0.548 |
| LAVp, mL | 13.85 (9.17,20.05) | 16.40 (11.27, 26.00)^*^ | 13.25 (9.63, 17.65) | 7.187 | 0.028 |
| adjusted^a^ | 12.21 (9.14,15.28) | 14.45 (10.57,18.33) | 15.22 (13.12,17.33) | 1.095 | 0.337 |
| LATEF, % | 35.85 (29.25,46.88)^*,†^ | 55.00 (42.25, 58.95)^*^ | 60.00 (57.28, 63.05) | 83.541 | < 0.001 |
| adjusted^a^ | 35.59 (30.33, 40.85) | 55.07 (50.79, 59.35) | 59.70 (57.67, 61.73) | 31.789 | < 0.001 |
| LAVaEF, % | 22.11 ± 13.27^*,†^ | 30.51 ± 12.89^*^ | 39.33 ± 10.91 | 28.560 | < 0.001 |
| adjusted^b^ | 22.31 (17.89, 26.74) | 30.73 (27.26, 34.21) | 39.11 (36.06, 42.16) | 15.632 | < 0.001 |
| LAVpEF, % | 19.40 ± 8.82^*,†^ | 29.09 ± 12.33^*^ | 33.45 ± 12.07 | 18.286 | < 0.001 |
| adjusted^b^ | 19.24 (14.95,23.54) | 28.92 (25.55,32.29) | 33.62 (30.65,36.58) | 13.202 | < 0.001 |
| LAEI, % | 55.77 (41.32, 88.71)^*,†^ | 119.81 (76.77, 145.50)^*^ | 149.99 (134.39, 170.81) | 84.731 | < 0.001 |
| adjusted^a^ | 55.33 (40.72,69.93) | 119.85 (102.79,136.91) | 149.33 (137.89,160.76) | 43.800 | < 0.001 |
| LASI | 0.97 (0.67, 1.42)^*^ | 0.64 (0.45, 0.87)^*^ | 0.18 (0.15, 0.21) | 140.172 | < 0.001 |
| adjusted^a^ | 0.96 (0.75,1.17) | 0.64 (0.54,0.74) | 0.19 (0.17,0.21) | 56.658 | < 0.001 |
| LACI, % | 36.27 (22.34, 42.22)^*,†^ | 24.23 (17.91, 33.09) | 23.94 (20.74, 28.98) | 12.955 | 0.002 |
| adjusted^a^ | 35.29 (29.74,40.84) | 23.06 (19.44,26.68) | 24.56 (22.77,26.34) | 7.635 | < 0.001 |
| ^*^*P* *<* 0.05 vs controls; ^†^ *P <* 0.05 vs LVEF ≥ 50% group.  ^a^ Adjusted median (95% CI) after adjusting for age by quantile regression;  ^b^ Adjusted mean (95% CI) after adjusting for age by analysis of covariance.  Abbreviations: AS = Aortic stenosis; LVEF = Left ventricular ejection fraction; LA = Left atrial; LAVmax = Left atrial maximal volume; LAVmin = Left atrial minimal volume; LAVpre = Left atrial pre-systolic volume; LAVI = Left atrial volume index; LASV = Left atrial stroke volume; LAVa = Left atrial active emptying volume; LAVp = Left atrial passive emptying volume; LATEF = Left atrial total emptying fraction; LAVaEF = Left atrial active emptying fraction; LAVpEF = Left atrial passive emptying fraction; LAEI = Left atrial expansion index; LASI = **Left atrial stiffness index**; LACI = **Left atrioventricular coupling index.** | | | | | |
|  |  |  |  |  |  |
|  |  |  |  |  |  |
