# Supplementary material for: Echocardiographic assessment of left atrial structure and function in severe aortic stenosis with preserved vs. reduced left ventricular ejection fraction
Source: Front Cardiovasc Med. 2026 May 21;13:1738702. doi: 10.3389/fcvm.2026.1738702 (PMC13233431; doi:10.3389/fcvm.2026.1738702)
Supplement: Supplementary file 3 [file Table3.docx]

| **Table S3. LA strain parameters in patients with severe AS and controls**,** mean ± SD.** | | | | | |
| --- | --- | --- | --- | --- | --- |
| LA strain parameters | LVEF < 50%  (n = 34) | LVEF ≥ 50%  (n = 65) | Controls  (n = 96) | *F* | *P* Value |
| LASr, % | 19.23±5.32^*,†^ | 23.64±6.31^*^ | 39.78±6.11 | 213.457 | < 0.001 |
| adjusted^a^ | 19.43(17.21,21.66) | 23.86(22.11,25.61) | 39.55(38.02,41.09) | 98.359 | < 0.001 |
| LASct, % | -9.47±5.18^*^ | -11.04±6.14^*^ | -23.58±8.55 | 99.356 | < 0.001 |
| adjusted^a^ | -9.48(-12.17,-6.79) | -11.05(-13.16,-8.94) | -23.57(-25.42,-21.71) | 36.740 | < 0.001 |
| LAScd, % | -9.76±5.47^*,†^ | -12.59±5.64^*^ | -15.81±5.90 | 15.703 | < 0.001 |
| adjusted^a^ | -10.25(-12.35,-8.14) | -13.10(-14.76,-11.45) | -15.29(-16.74,-13.83) | 6.331 | 0.002 |
| PALS, % | 19.39±5.28^*,†^ | 23.15±6.26^*^ | 39.51±5.81 | 224.489 | < 0.001 |
| adjusted^a^ | 19.79(17.63,21.94) | 23.57(21.88,25.27) | 39.09(37.60,40.58) | 98.283 | < 0.001 |
| ^*^*P* *<* 0.05 vs controls; ^†^ *P <* 0.05 vs LVEF ≥ 50% group.  ^a^ Adjusted mean (95% CI) after adjusting for age by analysis of covariance.  Abbreviations: AS = Aortic stenosis; LA = Left atrial; LVEF = Left ventricular ejection fraction; LASr = Left atrial reservoir strain; LASct = Left atrial contractile strain; LAScd = Left atrial conduit strain; PALS = Peak atrial longitudinal strain. | | | | | |
|  |  |  |  |  |  |
|  |  |  |  |  |  |
